# Between Habitats: Transfer of Phytopathogenic Fungi along Transition Zones from Kettle Hole Edges to Wheat Ears

**DOI:** 10.3390/jof9090938

**Published:** 2023-09-16

**Authors:** Marina Gerling, Grit von der Waydbrink, Gernot Verch, Carmen Büttner, Marina E. H. Müller

**Affiliations:** 1 Leibniz Centre for Agricultural Landscape Research (ZALF), Eberswalder Str. 84, 15374 Müncheberg, Germany; 2 Albrecht Daniel Thaer-Institute, Faculty of Life Science, Humboldt-Universität zu Berlin, 14195 Berlin, Germany

**Keywords:** *Alternaria*, *Fusarium*, kettle hole, moisture, semi-natural landscape element (NLEs), source of infection, transition zones, wheat

## Abstract

Kettle holes are able to increase the soil and air humidity around them. Therefore, they create a perfect habitat for phytopathogenic fungi of the genera *Fusarium* and *Alternaria* to develop, sporulate, and immigrate into neighboring agricultural fields. In our study, we establish transects from the edges of different kettle holes and field edges up to 50 m into the fields to analyze the abundance and diversity of pathogenic fungi in these transition zones by culture-dependent and culture-independent methods. However, in 2019 and 2020, low precipitation and higher temperatures compared to the long-time average were measured, which led to limited infections of weeds in the transition zones with *Fusarium* and *Alternaria*. Therefore, the hypothesized significantly higher infection of wheat plants next to the kettle holes by a strong spread of fungal spores was not detected. Infestation patterns of *Fusarium* and *Alternaria* fungi on weeds and wheat ears were spatially different. In total, 9 different *Fusarium* species were found in the transition zone. The species diversity at kettle holes differed from 0 to 6 species. The trend toward increased dryness in the northeast German agricultural landscape and its impact on the changing severity of fungal infections is discussed.

## 1. Introduction

Yield losses due to various factors are a major agricultural problem. Annually, up to 15–20% of agricultural yield losses are caused by fungal-associated diseases worldwide [1,2]. Wheat (*Triticum aestivum* L.) is one of the most economically important crops in the world [3], but unfavorably, wheat is highly susceptible to fungal infections. *Fusarium* and *Alternaria* fungi are responsible for prominent infections of various crops, including summer and winter wheat [4,5,6,7]. 

In the case of fungal diseases on wheat, *Fusarium* head blight (FHB) poses farmers with significant challenges: *Fusarium* infections, caused by up to 19 different *Fusarium* species [8,9,10,11], can lead to significant pre-harvest yield and through contamination of the kernels with mycotoxins [2,12,13], whereas *Alternaria* is mostly known to cause black and gray rot, and the black spot leaf disease [4].

With regard to fungal infection processes, remaining crop debris above and below the soil can harbor pathogenic fungi and support their development and sporulation, and offer them a habitat to overwinter [10,14,15,16,17,18]. Arable weeds were often underestimated, but are now recognized to play a major role in the inoculation of crops with pathogenic filamentous fungi [3,10,16,19,20,21,22,23]. They offer a broad host range for fungal pathogens, including *Fusarium* and *Alternaria*, and can act as both alternative and alternate hosts for these species [24]. However, compared to crop debris as a source of infection, there is little information available about arable weeds as reservoirs for pathogenic fungi [10,15,16,20,21,22,23]. One reason for this information gap could be that infections of most weeds are asymptomatic. Weeds often do not show symptoms of disease, even when they are highly infected. However, if weeds in arable lands act as reservoirs for fungal pathogens, the fungal spores may also immigrate from the non-cultivated hosts to the cultivated ones by wind, rain, and through transport by macro-organisms [25,26,27,28]. 

Weeds, which are part of every agricultural system, occur alongside the crops in the field season. Furthermore, weeds can grow permanently at (semi)-natural landscape elements (NLEs), like kettle holes or hedgerows in the field or at field margins, over several field seasons, because they are not harvested alongside the crops.

NLEs are responsible for borders, edges, and transition zones in arable lands, because they can divide the agricultural landscapes into different small-scale areas [29,30]. These spatial heterogeneities are uniquely important structures, especially on fields with a unilateral crop rotation, because NLEs can act as keystone structures [31] by offering several ecosystem services (providing habitat, access to water or enhanced moisture, food, and shelter) for numerous species of macro-organisms (e.g., ground beetles, breeding birds, amphibians, bees) and micro-organisms (e.g., fungi, bacteria) [32,33,34,35,36,37]. Small water bodies occurring in arable lands are often kettle holes, which are defined as natural ponds with <1 ha area [38]. These kettle holes act as specific NLEs by increasing the soil- and air moisture in the field and thereby influencing humidity-sensitive organisms in the transition zones, between the kettle holes and the adjacent agricultural field. Field edges, as semi-natural landscape elements, may also influence the abundance and diversity of pathogenic fungi in the neighboring fields (not by increasing the humidity).

The life cycle of filamentous fungi, like *Fusarium* spp., is highly influenced by moisture conditions in their living environment and *Fusarium* is known to be found more frequently in habitats with higher humidity [6,39]. As such kettle holes provide *Fusarium* species with a suitable habitat to overwinter or outlast times when their main host is absent [22].

Nevertheless, in Germany, there is a ban on the use of chemical applications next to kettle holes, because they are protected areas by law [40]. For this reason, the control of weeds with herbicides, or the control of fungal pathogens by fungicides next to kettle holes is prohibited. Thus, kettle holes act as a suitable habitat for annual and especially perennial weed plants to survive in homogeneous agricultural landscapes. 

Due to the combined effect of the different types of plants growing at the edges of NLEs, and the increased humidity originating from water-filled NLEs, kettle holes are regarded as suitable habitats for several macro-organisms (e.g., breeding birds, pollinators, ground beetles [27,36]), and also offer different living conditions and microhabitats for a wide variety of (pathogenic) fungi. Although kettle holes provide many ecosystem services, they are thought to enhance fungal development and infection of agricultural fields, especially in the transition zones between kettle holes and neighboring fields. Transition zones in our context are defined as areas where two adjacent structures (kettle hole, field, field edge) interact and may influence each other. For example, studies by Raatz et al. [30,41] describe yield decreases next to kettle holes up to 11 m (through reduced use of chemical preparations close to them) and higher infection of wheat leaves next to kettle holes.

Against this background, we hypothesize that kettle holes in arable lands influence the abundance and diversity of phytopathogenic fungi in adjacent wheat fields through (1) weeds growing permanently at the edges of kettle holes, acting as a reservoir for the growth and sporulation of these fungi, and (2) increased moisture in the transition zone between kettle hole and wheat field. We assume fungal abundance in the field to be highest directly next to kettle holes and to decrease with increasing distance.

For these aims, in July 2019 and 2020, we investigated transition zones originating from 20 different kettle into adjacent wheat fields. To test our hypothesis, we first analyzed the abundance and diversity of phytopathogenic fungi in and on arable weeds growing at the edges of kettle holes and field edges. Furthermore, we sampled wheat ears at 4 different sampling points along a transect from the edges (kettle hole/field edge) into the field up to 50 m. Field edges were used as a comparison because there were also weeds growing there, but there was no source of extra moisture. We analyzed the abundance and diversity of phytopathogenic fungi on the wheat ears to examine if the kettle holes acted as a reservoir for fungi, and supported the infection of the wheat plants growing near them.

## 2. Materials and Methods

### 2.1. Study Site 

The study site is located on farms within the Quillow catchment in the Uckermark in the north-east Brandenburg, Germany. The area is dominated by agricultural land use. Scientific investigations took place in the long-term research platform “AgroScapeLab Quillow” (Agricultural Landscape Laboratory Quillow, E 013°48′12″, N 53°21′59″) of the Leibniz Centre for Agricultural Landscape Research (ZALF) [42,43,44]. 

The topography of the study site is characterized by a hummocky landscape, massively reshaped during the Pleistocene [42,43], where small water bodies (<1 ha [45]), called kettle holes, are frequent semi-natural landscape structures. More than 1500 kettle holes are located in the “AgroScapeLab Quillow”. The land use types are composed of 74.4% agrarian fields, 10.4% grasslands, and 5.9% forest, and 1.4% of the area is covered by up to 40 kettle holes per km^2^, which occur in all of the land-use types previously mentioned [42]. 

The study was implemented in winter wheat fields where maize was the preceding crop. The examined fields belonged to commercial farms, so the crops were managed according to standard agricultural procedures and good professional practices.

### 2.2. Sampling Design

**Figure 1:**
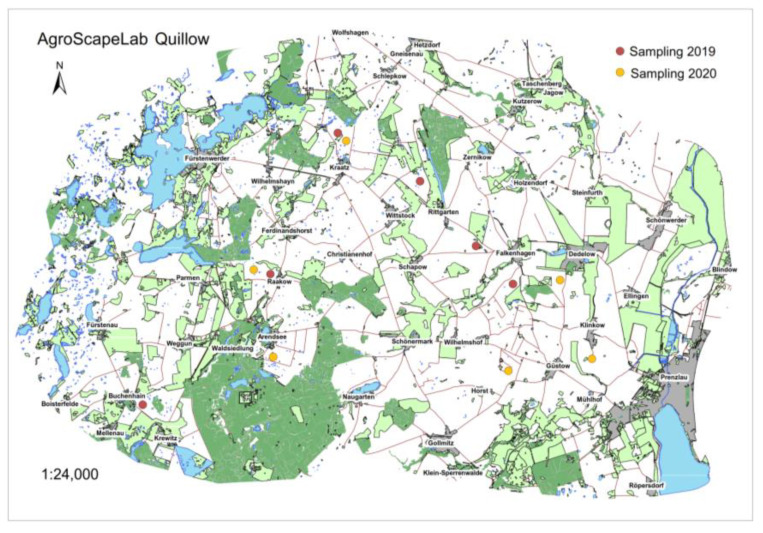
Spatial distribution of fields with kettle holes thereon, investigated in 2019 (red dots) and 2020 (orange dots) within the “AgroScapeLab Quillow” region.

The field sampling took place in two consecutive years (2019, 2020) on 6 different wheat fields at 10 different kettle holes in total each year, while 4 fields had 2 kettle holes on them. The location of these fields within the Uckermark region, Germany, is displayed in Figure 1.

Transects were set up at 10 kettle holes and 6 field margins into the adjacent field with sampling points at −1 m, 1 m, 5 m, 20 m, and 50 m. From the first sampling point at −1 m (kettle hole edge or field margin), we sampled three different (the most frequent) non-crop plants, which were taken in 1 square meter around this point. Both young and fresh as well as senescent and necrotic plant parts (if present) were included. The sample collection comprised of a total of 96 plant samples. At the other sampling points (1 m up to 50 m), wheat ears were sampled. 15 different wheat ears from each sampling point were randomly picked in a 0.5 m area around the sampling point and cut 2 cm below the ear. The samples were collected in crispac bags for weeds and paper bags for wheat ears and transported to the laboratory in cool boxes. The collected samples were stored at 4–6 °C until further investigations on the next day. Both weeds and wheat ears were analyzed by culture-dependent and culture-independent methods for the presence of filamentous fungi of the genera *Fusarium* and *Alternaria*. Furthermore, the species composition of *Fusarium* was analyzed. We sampled in July, 2 weeks after full flowering because wheat ears are most susceptible to fungal infections in the flowering and early ripening stages [46]. 

### 2.3. Laboratory Analyses 

#### 2.3.1. Culture-Dependent Method

For determination of the colony forming units (cfu) per gram of fresh matter for *Fusarium* (FUS_cfu/gFM) and *Alternaria* abundance (ALT_cfu/gFM), potato dextrose agar (PDA; Merck, Heidelberg, Germany) supplemented with chloramphenicol, and synthetic nutrient agar (SNA) [47] were used as described detailed by Leslie and Summerell [48]. Ten pieces (about 1 cm in length) of each weed plant sample (randomly selected pieces of leaves, stems, and/or flowers) were plated onto two PDA containing Petri dishes (diameter 9 cm): five pieces plated on each plate. Before plating, we weighted the plant samples (Kern 572-35; Kern&Sohn GmbH, Balingen-Frommern, Germany) to calculate the colony forming units to 1 g of plant fresh matter. 

Wheat samples were prepared as described above by using 10 kernels per sampling point from 10 different wheat ears (one kernel per ear), randomly chosen from the bottom, middle, and tip of the ear to calculate the infection rate of the wheat ears (counted colony forming units × 10). Plated samples, both grass samples and wheat samples, were incubated for 2 days at 24 °C in darkness and further 2 days under UV light (12 h UV light/12 h daylight) at room temperature to support the sporulation and coloration of the fungal mycelium. The colony-forming units of *Fusarium* and *Alternaria* were counted 2 days after the UV light treatment. 

Colonies of *Fusarium* were isolated onto a new PDA Petri dish and also sub-cultured onto SNA media for the morphological identification of the species. PDA was used to analyze the species by morphological aspects (growth rate and color of the mycelium), while SNA supported different *Fusarium* species in developing their species-specific macro- and microspore characteristics. Plates were treated as described above except with a longer UV light treatment (up to 10 days) depending on the growth rate of the mycelium. Isolated *Fusarium* fungi were identified at a species level using light microscopy (Jenaval, Carl Zeiss, Jena, Germany) and the identification was mainly based on macro- and micro-morphological characteristics described by Leslie and Summerell [48].

#### 2.3.2. Culture-Independent Method (qPCR Approach)

The remaining samples (weeds and wheat ears) were dried at 60 °C for at least 48 h. For further analyses by real-time quantitative polymerase chain reaction (qPCR), the dried samples were ground using a vibrating cup mill RS200 (Retsch, Haan, Germany) at 1300 rpm for 1.5 min for weeds, or 1000 rpm for 45 s. for wheat ears. Afterward, the ground material (250 mg for grasses or 50 mg for wheat ears) was carefully mixed and used for genomic DNA extraction according to the customized standard protocols of the following DNA extraction kits: NucleoSpin^®^ Soil Kit (MACHEREY-NAGEL GmbH & Co. KG, Düren, Germany) for weeds and DNeasy Plant Mini kit (QIAGEN GmbH, Hilden, Germany) for wheat ears. Once milled, the material was carefully mixed, and the DNA was extracted according to a customized standard protocol of the kits according to the manufacturer’s instructions for handling. The quantification of DNA gene copy numbers of *Fusarium* and *Alternaria* by a qPCR approach for both, weeds and wheat ears, with genus-specific primers was described in detail by Gerling et al. [22]. Fungal strains used for the preparation of the standard curves were stored in a culture collection of fungi of the working group “Fungal Interactions” at the Leibniz Centre of Agricultural Landscape Research Müncheberg. All qPCR assays contained negative controls, and all measurements were done in duplicate. The genome copy numbers were expressed in FUS_gcn/gDM (dry matter) for *Fusarium* and in ALT_gcn/gDM for *Alternaria* fungi. 

#### 2.3.3. Analyses of Deoxynivalenol (DON) and Zearalenone (ZEN)

The extraction of the mycotoxins for the analyses of deoxynivalenol (DON) and zearalenone (ZEN) was described in detail by Müller et al. [49], while the used HPLC method was described by Gerling et al. [23]. Each analysis was performed in duplicate. All toxin concentrations were calculated on the DM of the substrate (ng/g DM) and the toxin detection limits in the grains were 30 ng DON and 2 ng ZEN per gram of substrate DM.

### 2.4. Microclimate

In 2019, along all transects, microclimatic observation stations (Onset, HOBO, Bourne, MA, USA) were installed at 5 different distances (−1 m; 1 m; 5 m; 20 m; 50 m) to monitor air temperature and air humidity during the growing season of wheat plants (between April and June 2019—every 15 min.) [50]. In 2020, we monitored air temperature, air humidity, leaf wetness, and soil moisture during the growing season of wheat plants (between March and July 2020—every hour) at 4 different distances (1 m, 5 m, 20 m, 50 m) [51].

### 2.5. Statistics 

All statistical tests were performed using SPSS (IBM SPSS Statistics V 22.0). For the visualization of the gcn/gDM of fungal abundances as boxplots, a logarithmic transformation LOG (x + 1) was applied to the data of the culture-independent method (qPCR approach). The midline represents the median; the upper and lower limits of the boxes are the third and first quartile. The abundance data (cfu/gFM and gcn/gDM) was tested for normal distribution via Kolmogorov-Smirnov test. Differences in fungal quantities between the different *Fusarium* species (gcn/gDM) and mycotoxin accumulations (ng/g), as well as the microclimatic data, were compared by the Kruskal–Wallis test and Mann–Whitney-U test (*p* values < 0.05). 

## 3. Results

### 3.1. Comparing Transition Zones (Kettle Hole vs. Field Margin)

#### 3.1.1. Weeds at the Edges of Kettle Holes and at Field Margins

**Figure 2:**
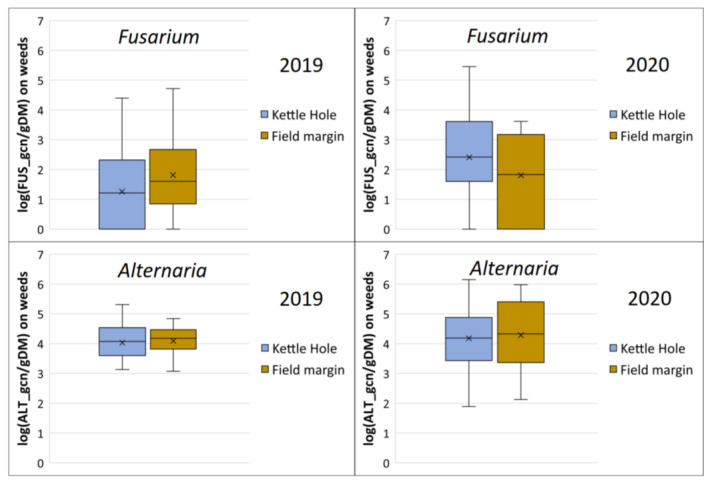
Abundances of *Fusarium* and *Alternaria* fungi at kettle holes (n = 10) and field edges (n = 6) in 2019 (end of June) and 2020 (beginning of July) expressed as LOG (x + 1) (gene copy numbers) of *Fusarium* (FUS_gcn/gDM) and *Alternaria* (ALT_gcn/gDM) on weed plants sampled in the AgroScapeLab Quillow. Data of all kettle holes and all field edges pooled together. The midline of the boxplots represents the median; the x is the mean value; the upper and lower limits of the boxes are the third and first quartile.

First, the abundances of *Fusarium* and *Alternaria* fungi (gcn/gDM) on the weed plants sampled at the edges of the kettle holes and at the field margins were compared; however, the data analyzed shows no statistically significant differences (* *p* < 0.05) in fungal infestation, both for *Fusarium* and *Alternaria* in the two years investigated (Figure 2). 

It is noticeable, however, that we detected a higher abundance of *Alternaria* in total, both at the kettle holes and at the field margins, in contrast to *Fusarium*. Furthermore, the abundance of *Fusarium* fungi is more widely scattered when all field and kettle-hole edges examined were considered: *Fusarium* samples showed a larger standard deviation. We analyzed samples with 0 gcn/gDM up to samples with 200,000 gcn/gDM and more (286,571 gcn/gDM was the highest measured value). Compared to this, the abundance of *Alternaria* was more evenly distributed over all field- and kettle-hole edges.

Both in 2019 and 2020, gramineous weeds were more infected with *Fusarium* fungi than herbaceous plants. For example, in 2019, 98% of all *Fusarium* gcn/gDM were found on grasses. *Alternaria* fungi showed a different pattern: herbaceous plants were more infected with *Alternaria* than grassy weeds. In 2020, we detected 99,462 *Alternaria* gcn/gDM on gramineous weeds and 200,647 gcn/gDM on herbaceous plant samples.

#### 3.1.2. Wheat Ears along the Transition Zones 

**Figure 3:**
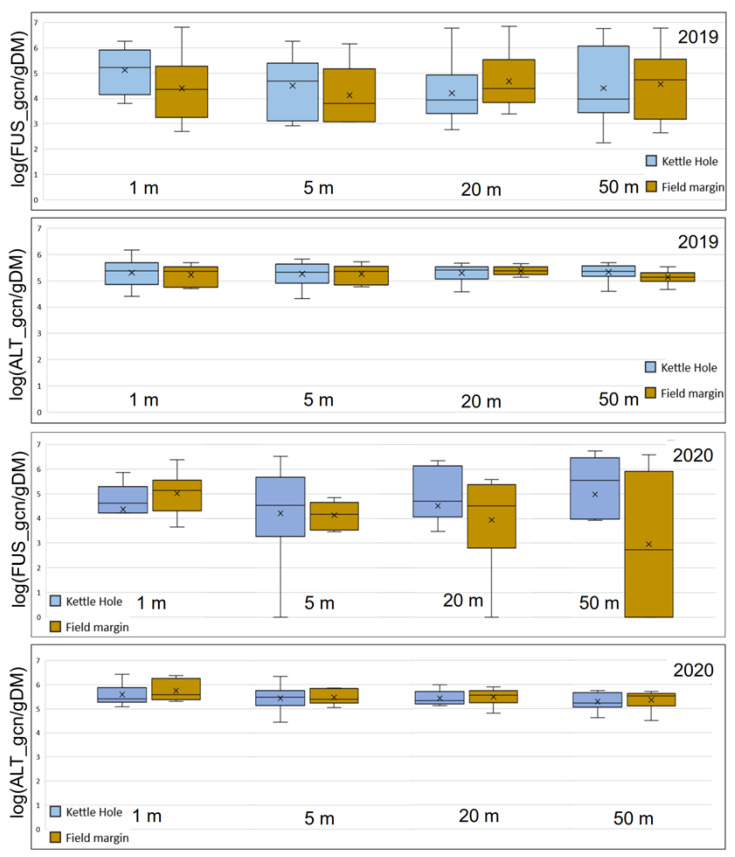
Mean value of the abundance of *Fusarium* and *Alternaria* fungi expressed as LOG (x + 1) (gene copy numbers) of *Fusarium* (FUS_gcn/gDM) and *Alternaria* (ALT_gcn/gDM) on wheat ears at the kettle holes (n = 10) and the field margins (n = 6) at the sampling points at 1 m, 5 m, 20 m, and 50 m in 2020, sampled in the AgroScapeLab Quillow. The midline of the boxplots represents the median; the x is the mean value; the upper and lower limits of the boxes are the third and first quartile.

Furthermore, the abundances of *Fusarium* and *Alternaria* fungi (gcn/gDM) on the wheat ears along the transition zone between the semi-natural landscape element and the field, were compared as shown in Figure 3.

Regarding *Fusarium*, no significant differences in relation to the distance from the edges to the middle of the field (50 m) were found: not at the transects from the kettle holes into the field, nor at the transects originating from the field margins. We only were able to detect slightly higher abundances at the sampling points at 1 m in 2019. Also, the abundance of *Fusarium* on the wheat ears growing near the kettle holes was roughly equal to the abundance of *Fusarium* on the wheat ears next to the field edges. 

The same pattern was detected for *Alternaria* fungi. However, in total, slightly higher abundances of *Alternaria* were found on the wheat ears analyzed, compared to *Fusarium*.

It is noticeable that the wheat ears were more infected with both pathogenic fungi, *Fusarium* and *Alternaria* than the weed samples from the kettle holes and field edges. This difference was more pronounced in the *Fusarium* infestation than in the *Alternaria* infestation. *Alternaria* infestation was 3× fold higher on the wheat ears, while *Fusarium* infestation was 83× fold higher. The mean value of *Fusarium* on the weeds (field edge and kettle hole edge) was 7670 gcn/gDM, compared to 639,875 *Fusarium* gcn/gDM on wheat. The abundance of *Alternaria* on the wheat ears was 358,951 gcn/gDM, while we only detected a mean value of 99,349 *Alternaria* gcn/gDM on the weed plants. Regarding *Fusarium*, once again, the measured abundances were much more heterogeneous (most mean values between approximately 2000 and 400,000 gcn/gDM) over all fields and kettle hole edges examined compared to *Alternaria*. The abundances of *Alternaria* were very consistent: most of the measured values were between approximately 100,000 and 400,000 gcn/gDM.

#### 3.1.3. Air Humidity at the Kettle Holes and Field Margins

**Figure 4:**
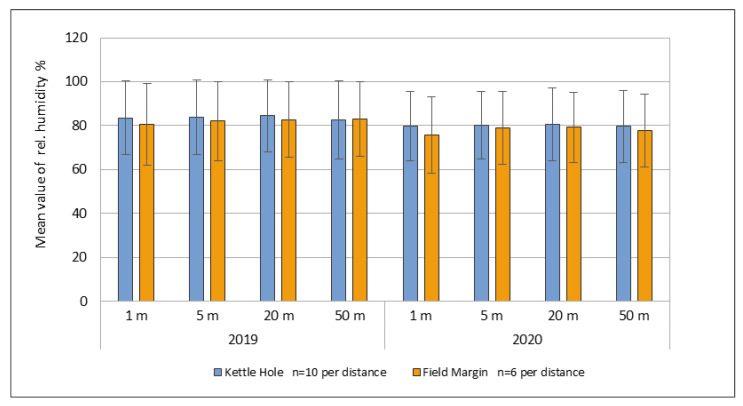
Mean value of the relative air humidity along the transition zones (kettle holes, field margin) at the sampling points (1 m, 5 m, 20 m, 50 m) in May and June of the years investigated (2019 and 2020).

The microclimatic measurements of the relative humidity in the months of May and June have shown no significant differences along the transition zones from the kettle hole into the field and from the field edges into the field (Figure 4).

Only at the sampling point 1 m minimal differences could be measured: 2% higher air humidity in the immediate vicinity to the kettle holes in 2019 as well as in 2020. Furthermore, also the distance to the kettle hole edge did not represent a difference. Regarding the weather data of the two years investigated, we observed two dry years compared to the long-term average: long time average precipitation sum from 1992–2015: 495 mm, precipitation sum in 2019: 459.8 mm, and precipitation sum in 2020: 423.8 mm. Due to this decrease in precipitation, an influence of the kettle holes on the abundance of *Fusarium* and *Alternaria* fungi in the field due to the water supply could not be detected.

### 3.2. Diversity of kettle Holes in 2020

#### 3.2.1. Diversity in Size, Water Performance, and Vegetation Type 

In the following chapter, different parameters of the kettle holes investigated in 2020 are presented to exemplarily show the differences between these 10 kettle holes. 

**Figure 5:**
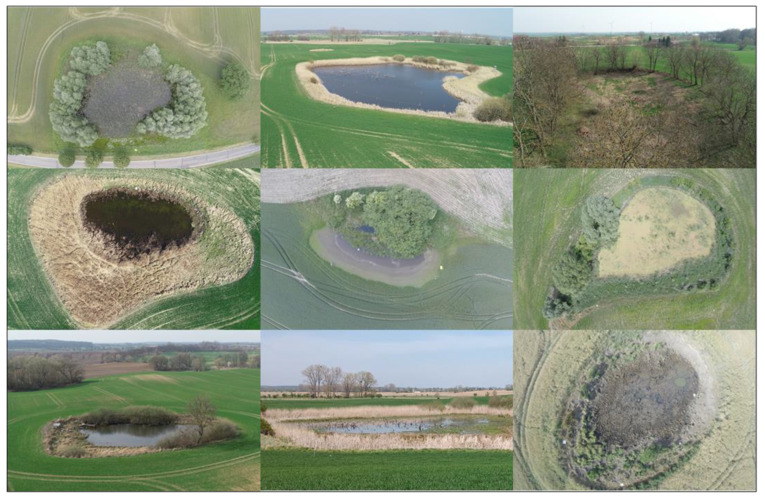
The diversity of 9 different kettle holes investigated in the AgroScapeLab Quillow in 2019 and 2020. The picture shows both, water-filled and non-water-filled kettle holes and the differences in the edge vegetation: trees, grasses, herbaceous plants (pictures copyright: Marlene Pätzig).

Figure 5 shows that the NLE “kettle hole” is very diverse. Some kettle holes are permanently water-filled (not in very dry years), while others temporarily dry out. Also, kettle holes are divided into two groups: the storage type, which stores all the incoming water, and the overflow type, which overflow after heavy rain events. Additionally, kettle holes differ considerably in size and type of edge vegetation.

Table 1 clearly shows how the 10 different kettle holes studied in 2020 differed from each other. They have strong differences in size (458 m^2^ up to 5762 m^2^), vegetation type and water performance, with the last parameter being the particularly most interesting one for the study of phytopathogenic fungi. 

In 4 (nr. 163, 172, 808, and 1192) out of 10 kettle holes, no water flow was detected for the entire year of 2020. In 2 other kettle holes (nr. 166 and 1193), a water performance was only measured in one month of 2020 (February). For nr. 182, 529, 807, and 1772 a water flow was detected for more than 3 months (4 up to 11), while kettle hole 529 was water filled for almost the whole year (January–November). 

The differences in the water performance of the kettle holes offered *Fusarium* and *Alternaria* fungi very different living conditions at the edges of the different kettle holes. However, due to the overall low water filling of most kettle holes in both study years, it must be concluded that increased humidity could only have existed in the immediate vicinity of the kettle holes (if at all). The water performance was not sufficient to cause more humid conditions in the air and soil around the kettle holes in 2019 and 2020, thus explaining the similar air humidity values shown in Figure 4. Moreover, the vegetation type can influence the abundance and diversity of the fungal communities living at the kettle holes. Plants from the family *Poaceae* were more likely to be infected by high abundances of filamentous fungi of the genera *Fusarium* and *Alternaria* than other arable weeds.

#### 3.2.2. Differences in the Abundance of Phytopathogenic Fungi, Mycotoxin Concentration, and Species Diversity

Table 2 shows the fungal abundance of *Fusarium* and *Alternaria* fungi detected on the wheat ears in the transition zones from the kettle holes edges into the wheat field by qPCR method at 1 m, 5 m, 20 m, and 50 m. Furthermore, the mycotoxin contamination of the wheat kernels at harvest, caused by a *Fusarium* infection (DON and ZEN), was measured for the same sampling points. 

We were not able to detect significant differences, both in the fungal abundance (*Fusarium* and *Alternaria*) and in the mycotoxin contamination of the grain. We expected decreasing FUS_ and ALT_gcn/gDM and also decreasing mycotoxin contamination in the transition zones with increasing distance to the kettle holes, but were not able to confirm this pattern with our data. For example, at five kettle holes, the highest *Fusarium* abundance was analyzed at 50 m, the sampling point the most far away from the source of infection (kettle hole edge), with values up to 5,479,713 *Fusarium* gcn/gDM. At the same kettle holes (1192), only 426,464 *Fusarium* gcn/gDM were measured at the sampling point 1 m. Kettle hole 529 was 11 months filled with water but did not show the highest abundance of *Fusarium* fungi. The abundance of *Fusarium* varied between 0 and 5,479,713 gcn/gDM. Every kettle hole seems to show a different pattern in the abundance of *Fusarium*.

Regarding *Alternaria*, the fungal abundances were more evenly distributed when all transition zone distances were considered.

Table 2 also shows very low mycotoxin contamination in total, both at the sampling points next to the kettle holes and the sampling points 50 m away from the edges. Only one sample exceeded the toxin regulations set by the European Union (1250 ng/g): the DON concentration at one sampling point at kettle holes was 1735.4 ng/g. The EU maximum limit for ZEN (100 ng/g) was not exceeded by any sample. At 2 kettle holes (nr. 172, 182) we did not measure any mycotoxins, neither DON nor ZEN, and at kettle hole nr. 807 and 808 we only detected a mycotoxin concentration at one sampling point (11.6 ng/g DON at sampling point 20 m). We measured the relative air humidity (%) (Figure 4) at the 10 different kettle holes at all sampling points (1 m up to 50 m) in May and June. We did not detect an increase in the air humidity at any of the edges of the kettle holes, as we expected. We detected values between 72% and 88%. 

**Figure 6:**
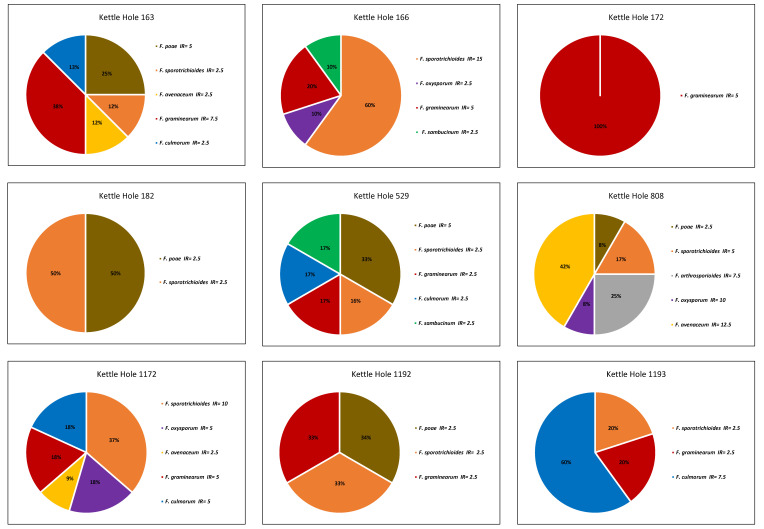
Infection rate of different *Fusarium* species (species diversity) on wheat ears sampled at 9 different kettle holes in the AgroScapeLabs Quillow, analyzed by a culture-dependent method. All sampling points in the transition zones pooled together (n = 4).

Figure 6 shows 9 out of 10 of the observed transects because at one kettle hole (807) we did not detect any *Fusarium* fungi. Also, the abundance of *Fusarium* in the other fields was low: measured infection rates were between 30 CFU/100 kernels and 5 CFU/100 kernels. 

In total, we isolated eight different *Fusarium* species in all of the transition zones examined: *F. sporotrichioides* (n = 9 fields), *F. graminearum* (n = 7), *F. poae* (n = 5), *F. culmorum* (n = 4), *F. avenaceum* (n = 3), *F. oxysporum* (n = 3), *F. sambucinum* (n = 3) and *F. arthrosporioides* (n = 1). Species diversity shows differences from one field to another. From kettle hole nr. 163, we isolated the highest amount of different *Fusarium* species (six), while at kettle hole nr. 172, we only found one species. At the other kettle holes, the species diversity differed from two to five different species. Regarding the different *Fusarium* species, the species composition seemed to be arbitrary. However, we isolated *Fusarium sporotrichioides* from eight out of nine kettle holes, and *Fusarium graminearum* occurred more often than other *Fusarium* species (seven out of nine). Against our expectations, the most humid kettle hole (nr. 529) did not show the highest species diversity. On the contrary, we detected six different *Fusarium* species at kettle hole nr. 163 (one of the ones which was dried out the whole year).

## 4. Discussion

Several previous studies [20,21,22,24,52] have demonstrated that weeds along field margins and edges of NLEs are very favorable habitats for the development, growth, and sporulation of *Fusarium* and *Alternaria* fungi. More specifically, weeds around kettle holes are an attractive (overwintering-) habitat for pathogenic fungi. From these overwintering sites, spores are then able to spread via wind, plant-to-plant contact, rain splash, and mobile linkers [25,26,27,28,53]. Both the humidity and high abundance of different weed plant species offer a perfect habitat for these fungal genera to overwinter on these hosts when the main crops are absent in autumn and winter. Despite this, these fungi can also colonize the growing and maturing weeds during the summer season [52,54].

However, in the months from May to July, the settlement and sporulation of fungi on weeds seem to depend strongly on weather conditions. In contrast to Gerling et al. and their study on weed infestation by fungi in autumn and winter [22], in the present study, lower colonization of weeds was found both at field edges and at kettle hole edges compared to the rainy autumn/winter in 2019/2020. For example, the mean value of *Fusarium* on grassy weeds in 2019 (field edge and kettle hole edge) in this study was 8056 gcn/gDM, compared to 12,703 *Fusarium* gcn/gDM on gramineous weeds in autumn/winter 2019/2020. The abundance of *Alternaria* on these overwintering weeds was even higher: 443,884 gcn/gDM in autumn/winter, while we only detected a mean value of 17,156 *Alternaria* gcn/gDM on weed plants in summer 2019. 

We attribute this to the very dry and warm summers of our study years 2019 and 2020: there was 35 mm (2019) and 71 mm (2020) less precipitation and increased temperature from May to August (appr. 1.5 °C) compared to the long-term average of years 1992–2015. These weather conditions, which were unfavorable for the present study, not only resulted in a likely lower infection rate of weeds, but also negated the special conditions at and around the kettle holes. 

Kettle holes are small, often water-filled sinks, occurring in all landscapes and formed during the ice age [38]. We did not detect the increased humidity that we hypothesized would be present in a zone close to the kettle holes to help encourage fungal development in this area. The low precipitation and elevated temperatures in 2019 and 2020 led to the fact that most of the kettle holes investigated were completely or partially dry, and the lack of water in the kettle holes did not then lead to increased humidity at the edge of the kettle holes.

This trend toward increased dryness in the northeast German agricultural landscape has already been observed since 2017 [55] and has strongly affected the water availability in kettle holes. In this respect, our hypothesis of colonization of weeds with *Fusarium* and *Alternaria* fungi and a pronounced dispersal of spores from the edges of kettle holes to the surrounding wheat ears was strongly limited by the weather effects. Both processes, the development of the fungi in a humid environment as well as spore dispersal, seem to be affected by the prevailing weather conditions [6,13,56]. Our hypothesis was based on the fact that we expected significantly higher values in soil and air moisture starting from kettle hole edges and decreasing into the field. We could not prove this in either of the 2 years under investigation. Thus, the non-significant differences in the colonization of the weeds at the field edge and at the kettle hole edge can be explained: there were no different moisture conditions at these two positions. Moist conditions would not only be a decisive advantage for the development of fungi, but also spore development, spore release, and spore dispersal benefit from increased humidity in the plant stand. This has already been proven in studies by Landshoot et al. [10] and Backhouse et al. [57].

It was remarkable that the abundance of *Fusarium* and *Alternaria* fungi in wheat ears in total was higher compared to the abundance of these fungi in weeds at the edges (and all positions) along the transition zones between the kettle hole and the wheat field. The mean value of *Fusarium* on the weeds (field edge and kettle hole edge) was 7670 gcn/gDM, compared to 639,875 *Fusarium* gcn/gDM on wheat. The abundance of *Alternaria* on wheat ears was 358,951 gcn/gDM, while we only detected a mean value of 99,349 *Alternaria* gcn/gDM on weed plants. It seems likely that there was only a limited spread of spores from weeds at the edge of kettle holes into the wheat field or–after a week of spore fly–the immigrated spores found a suitable habitat and favorable environmental conditions on wheat ears to develop there more strongly than on weeds. This can be assumed if the fungi infect wheat grains at the time of flowering and *Fusarium* fungi were able to develop more strongly inside the grain than on its surface. However, the low concentration of mycotoxins in the wheat grains indicates against this assumption. 

Other infection routes in the wheat field besides the transmission of spores from the weeds also need to be discussed. It is known that maize stubble can be a long-lasting source of infection and that spores living on these residues can spread to ears by rain splash on the growing wheat plant [58,59,60]. Further studies are necessary to clarify if and how airborne transmission from more distant sources of infection (or through mobile linkers) has contributed to the high abundance of *Alternaria* and *Fusarium* fungi in wheat ears. Air samples above the wheat field and the kettle hole edge should be included in investigations.

In the present study, we also found a higher and more equal *Alternaria* abundance compared to *Fusarium*. The infection by these necrotrophic fungi varied remarkably at all positions along the transects into the wheat fields in both years of investigations. Other studies have also shown a rather small-scale heterogeneity for the occurrence of *Fusarium* fungi and their mycotoxins in wheat fields [49]. The present results confirm once again that *Fusarium* tends to occur in hot spots and is heterogeneously distributed on a small scale in terms of both abundance and diversity. In contrast, *Alternaria* fungi showed a more uniform distribution, which can be confirmed in the present study by the low variance of the gcn along the transects in both study years. *Alternaria* prefers warm and drier living conditions [6], and besides their pathogenic phase, have a very pronounced saprophytic lifestyle, which benefits them, especially during the ripening of the grain ear. It would be interesting to study the population structure of this genus as well as to discover specific species diversity and draw conclusions on ecological preferences and behavior.

The advancing climate change is expected to cause a shift in the population of pathogenic fungi in crop plants [7,61,62]. *Fusarium* infestation will likely decrease, as they require constant high moisture for their development [6,63]. Due to less precipitation and longer and more severe dry periods, this humid environment is increasingly not available. This scenario was also observed at the kettle holes examined here, where most of them did not carry water in summer, as observed in the years before this study [55]. Therefore, less *Fusarium* infestation than expected was found on the weeds at the kettle hole edge. However, a decrease in *Fusarium* occurrence can only be expected as long as wheat fields are not irrigated artificially [23] or extreme rainfalls occur during the flowering stage of cereals. Most pathogenic fungi need moisture and heat for their development, but in some cases, droughts and high temperatures can be favorable: For example, at the end of this century, brown rust infections in wheat will start 20 days earlier and end 40 days later, with no infection-free summer period [64]. Chakraborty et al. [65] found that the increasing crop biomass and the number of infection cycles over more growing days caused by climate change will produce large rust populations. Also, increased concentrations of O3 can damage plant tissue and favor the development of necrotrophic pathogens such as *Botrytis cinerea* [66]. Such impacts can include changes in the predominant disease present on a crop, changes in the range or severity of epidemics, or the introduction of new diseases to a region [67,68,69].

Regarding future investigations, detailed information about the behavior of fungal pathogens living on NLE is of great importance for farmers. This implies that the investigations presented here will be repeated in further years. Our hypothesis remains valid and should be re-examined in wetter years with increased air and soil moisture and increased water availability in the kettle holes. In general, a better understanding of the fungal behavior in the NLE habitats helps farmers to make better predictions of suspected infections, thereby they can react quicker regarding their agricultural management, especially in the context of a changing climate. Abiotic stressors (e.g., increased temperature, salinity or carbon dioxide concentrations, and changes in water availability) will increase with climate change, and pose challenges to farmers. They already changing their practices while cultivating wheat. They try to avoid high temperatures during the grain-filling period by sowing the wheat earlier than usual. However, this practice has the disadvantage of (fungal) diseases having a longer period for development and infection of the crops. In the case of kettle holes, we recall the great importance of these semi-natural landscape elements for various ecosystem services like providing water and food sources, and as a habitat and shelter for different macro- and microorganisms [36,45,70,71]. Regarding the disservice and resulting trade-offs, we recommend mowing the edges of kettle holes at least once a year, in years with more precipitation several times, and removing plant residues from fields to prevent disease transfer to surrounding crop plants.

## Figures and Tables

**Table 1:** Kettle hole number, water permanence, area, and vegetation type of the 10 different kettle holes investigated in 2020.

Kettle Hole Number	Number of Months with Water (Water Performance)	Area m^2^ (Water Body and Edge Zone)	Vegetation Type *	*Poaceae* (%)
163	0	458	E-RCG/FL	25
166	1 (February)	1156	FR-RCG	33
172	0	4383	FR-R/RCG/N	18
182	4 (February–May)	836	W/FR-SW/R	22
529	11 (January–November)	2718	O-R	n/a
807	7 (January–July)	2375	E-SW/S/N	n/a
808	0	5762	FR-SW/N	n/a
1172	6 (January–June)	1367	E-SW	n/a
1192	0	851	FR-RCG/S/N	52
1193	1 (February)	775	E-SW	65

* E—edge type; FR—full reed type; O—open type; W—wood type; FL—flood lawn vegetation; N—nitrophilous perennials; R—reed; RCG—reed canary grass; S—sedges; SW—shore woods.

**Table 2:** Kettle hole number, distance from kettle hole edge into the wheat field (transition zone), gene copy numbers of *Fusarium* and *Alternaria*, and mycotoxin contamination (DON, ZEN) of wheat ears.

Kettle Hole No.	Distance	ALT_gcn/gDM	FUS_gcn/gDM	Zearalenone ZEA ng/g (ppb)	Deoxynivalenol DON ng/g (ppb)
163	1 m	1,535,720	733,597	0.0	249.4
163	5 m	438,579	3,354,345	0.0	177.6
163	20 m	202,472	1,216,214	not analyzed	not analyzed
163	50 m	89,285	2,477,367	0.0	1735.4
166	1 m	610,140	152,039	0.0	174.7
166	5 m	862,397	61,803	0.0	218.0
166	20 m	215,439	1,952,588	0.0	273.9
166	50 m	560,422	46,854	0.0	489.4
172	1 m	277,195	16,288	0.0	0.0
172	5 m	490,698	421,384	0.0	0.0
172	20 m	148,134	49,350	0.0	0.0
172	50 m	160,191	9837	0.0	0.0
182	1 m	190,607	75,010	0.0	0.0
182	5 m	27,883	0	0.0	0.0
182	20 m	153,886	58,455	0.0	0.0
182	50 m	123,957	8518	0.0	0.0
529	1 m	120,263	26,019	0.0	0.0
529	5 m	167,439	1599	0.0	245.5
529	20 m	128,847	2,250,616	0.0	246.2
529	50 m	434,664	3,770,598	0.0	134.1
807	1 m	249,016	34,926	0.0	0.0
807	5 m	319,805	3039	0.0	0.0
807	20 m	562,848	0	11.6	0.0
807	50 m	363,905	54,568	0.0	0.0
808	1 m	186,780	0	0.0	0.0
808	5 m	153,190	1977	0.0	0.0
808	20 m	326,597	18,575	0.0	134.9
808	50 m	121,039	0	0.0	0.0
1172	1 m	246,837	49,784	0.0	74.9
1172	5 m	99,134	114,845	0.0	134.7
1172	20 m	174,270	32,028	0.0	0.0
1172	50 m	40,805	2,662,938	0.0	198.9
1192	1 m	439,399	426,464	0.0	273.8
1192	5 m	281,322	18,649	0.0	276.8
1192	20 m	491,626	2929	0.0	360.5
1192	50 m	178,920	5,479,713	0.0	213.7
1193	1 m	2,725,250	16,252	57.8	110.6
1193	5 m	2,237,356	699,159	0.0	80.8
1193	20 m	945,137	51,578	8.4	121.2
1193	50 m	471,937	2,264,873	0.0	137.1

## Data Availability

The data presented in this study are available on request from the corresponding author.
